# Clinical and cost-effectiveness analysis of early detection of patients at nutrition risk during their hospital stay through the new screening method CIPA: a study protocol

**DOI:** 10.1186/s12913-017-2218-z

**Published:** 2017-04-20

**Authors:** José Pablo Suárez-Llanos, Néstor Benítez-Brito, Laura Vallejo-Torres, Irina Delgado-Brito, Adriá Rosat-Rodrigo, Carolina Hernández-Carballo, Yolanda Ramallo-Fariña, Francisca Pereyra-García-Castro, Juan Carlos-Romero, Nieves Felipe-Pérez, Jennifer García-Niebla, Eduardo Mauricio Calderón-Ledezma, Teresa de Jesús González-Melián, Ignacio Llorente-Gómez de Segura, Manuel Ángel Barrera-Gómez

**Affiliations:** 1 Endocrinology and Nutrition Department, Hospital Universitario Nuestra Señora de Candelaria, Ctra. Del Rosario 145, Santa Cruz de Tenerife, 38010 Spain; 2 Canary Foundation for Health Research (FUNCANIS) Evaluation Service of the Canary Health System (SESCS), Research Network on Health Services Chronic Disease (REDISSEC), Canary Center for Biomedical Research (CIBICAN), Santa Cruz de Tenerife, Spain; 3 General and digestive surgery Department, Hospital Universitario Nuestra Señora de Candelaria, Santa Cruz de Tenerife, Spain; 4 Internal Medicine Department, Hospital Universitario Nuestra Señora de Candelaria, Santa Cruz de Tenerife, Spain; 5 Hospital Universitario Nuestra Señora de Candelaria, Santa Cruz de Tenerife, Spain

**Keywords:** Nutrition assessment, Malnutrition, Inpatients, Body composition, Anthropometry, Cost-benefit analysis, Mass screening, Quality of life

## Abstract

**Background:**

Malnutrition is highly prevalent in hospitalized patients and results in a worsened clinical course as well as an increased length of stay, mortality, and costs. Therefore, simple nutrition screening systems, such as CIPA (control of food intake, protein, anthropometry), may be implemented to facilitate the patient’s recovery process. The aim of this study is to evaluate the effectiveness and cost-effectiveness of implementing such screening tool in a tertiary hospital, consistent with the lack of similar, published studies on any hospital nutrition screening system.

**Methods:**

The present study is carried out as an open, controlled, randomized study on patients that were admitted to the Internal Medicine and the General and Digestive Surgery ward; the patients were randomized to either a control or an intervention group (*n* = 824, thereof 412 patients in each of the two study arms). The control group underwent usual inpatient clinical care, while the intervention group was evaluated with the CIPA screening tool for early detection of malnutrition and treated accordingly.

CIPA nutrition screening was performed upon hospital admission and classified positive when at least one of the following parameters was met: 72 h food intake control < 50%, serum albumin < 3 g/dL, body mass index < 18.5 kg/m^2^ (or mid-upper arm circumference ≤ 22.5 cm). In this case, the doctor decided on whether or not providing nutrition support.

The following variables will be evaluated: hospital length of stay (primary endpoint), mortality, 3-month readmission, and in-hospital complications. Likewise, the quality of life questionnaires EQ-5D-5 L are being collected for all patients at hospital admission, discharge, and 3 months post-discharge. Analysis of cost-effectiveness will be performed by measuring effectiveness in terms of quality-adjusted life years (QALYs). The cost per patient will be established by identifying health care resource utilization; cost-effectiveness will be determined through the incremental cost-effectiveness ratio (ICER). We will calculate the incremental cost per QALY gained with respect to the intervention.

**Discussion:**

This ongoing trial aims to evaluate the cost-effectiveness of implementing the malnutrition screening tool CIPA in a tertiary hospital.

**Trial registration:**

Clinical Trial.gov (NCT02721706).

First receivevd: March 1, 2016

Last updated: April 8, 2017

Last verified: April 2017

## Background

Hospital malnutrition (HM) is a frequent condition in developed countries; its prevalence ranges from 25 to 50%, depending on the study population and the detection method [1–4]. This type of malnutrition is associated with the patient’s underlying condition, so that it may be triggered either by the disease-related symptoms (dysphagia, vomiting, diarrhea, malabsorption, etc.), thereby reducing food intake or increasing losses, or by an associated, increased caloric and protein requirement that the patient cannot cover.

Malnutrition in hospitalized patients leads to an exacerbated clinical course, with additionally emerging comorbidities and worsened functional capacity, which in turn lead to a longer hospital stay, increased mortality and health care costs [3–5]. It is, therefore, essential to detect these patients using nutrition screening techniques at hospital admission and thus try to improve their prognosis through nutrition therapy.

HM is as frequent as underrated, so that only few European countries and hospitals apply nutrition screening on admission. In the Spanish multicenter study PREDYCES, where screening was performed using Nutritional Risk Screening 2002 (NRS 2002), 23% of the hospitalized patients were found to be malnourished [6]. In addition, that malnourished subgroup underwent an aggravated clinical course during their hospital stay [2]. Based on these results, nutrition screening pilot projects are now being implemented in different hospitals throughout the country.

There is no gold standard for hospital nutrition screening, despite the high number of existing screening tools. It is, therefore, best to incorporate one that can be applied according to the hospital characteristics and that primarily identifies patients with the poorest clinical prognosis. In the University Hospital *Nuestra Señora de Candelaria* (HUNSC), Santa Cruz de Tenerife, Spain, the nutrition screening method CIPA (control of intakes, proteins, anthropometry) was designed by employing tools commonly used in clinical practice.

When CIPA screening was compared with Subjective Global Assessment (SGA) [7, 8] — the standard method for validation, nonetheless not a gold standard — in patients with a non-surgical pathology, those who resulted positive by CIPA screening had a longer mean stay than patients with a negative result (19.53 vs 12.63 days; *p* <0.001); this data was not detected by SGA screening. On the other hand, patients identified as positive by either method comprised more cases of death both in hospital (7.6% vs 1.4%, *p* = 0.026; CIPA screening) as well as by the first month post-discharge (12.7% vs 3.5%, *p* = 0.012) [9]. Therefore, in this group of patients, the CIPA screening tool detected those with a comparably worsened clinical course more efficiently than the SGA method.

Although malnourished patients have a worse prognosis and generate higher costs than the rest, the cost-effectiveness of performing a hospital nutrition screening is not sufficiently studied. Only few studies have performed an economic evaluation of a malnutrition screening in hospitalized patients. Kruzienga HM et al. analyzed the implementation of the screening method SNAQ (Short Nutritional Assessment Questionnaire) and concluded that its implementation was cost effective, mainly in the fragile patient [10]. The NICE guidelines on Nutrition Support in Adults [11] indicated that screening is likely to be cost effective but highlighted a high degree of uncertainty due to “the weakness in the methodologies and designs of identified studies.”

This study aims to shed light onto this area and study the clinical and economic impact through a randomized, controlled trial on the implementation of a nutrition screening method, in this particular case the CIPA method, in a tertiary hospital.

## Methods

### Trial design

This study is a two-armed, randomized, controlled trial, comprising an intervention arm, where patients were screened for malnutrition using CIPA at hospitalization, and a control arm, where patients were not screened but diagnosed according to standard clinical practice.

### Subjects

Patient inclusion criteria:

1. Patient admitted to the hospitalization ward of Internal Medicine or General and Digestive Surgery of the HUNSC.
2. Formal consent to participate in the study.
3. Age of 18 or more years.

Patient exclusion criteria:

1. Patient treated with nutrition support before finishing the CIPA screening procedure.
2. Patient transferred from other ward.
3. Patient with an expected length of stay of less than 72 h.
4. Unfeasible CIPA screening for whatever reason.
5. Patient with poor short-term prognosis.
6. Bed assignment at hospital admission non-randomized.
7. Patient participating in other research study.
8. Pregnancy.

### Setting and recruitment

Patients were recruited in the hospitalization area of the HUNSC, in the wards of Internal Medicine and General and Digestive Surgery. These departments were chosen for integrating the patients with the highest clinical variability and comorbidity. Recruitment was conducted in two phases: the first, at the time of hospitalization, in which the patient was confirmed to meet the inclusion or exclusion criteria and asked to sign the informed consent, thereby becoming potentially eligible. The second phase took place at 72 h, when the result of the CIPA nutrition screening was obtained, point at which the patient was verified not to have received nutrition treatment by that time and eventually became part of the sample. Otherwise, the patient was excluded from the study according to criterion 1).

### Random assignment

Both scheduled and emergency admission patients were allocated by the department of hospital admission to one of the participating wards, following the randomization plan, which was generated by simple random sampling and delivered by the data manager of the project. This randomization facilitated trial execution and prevented protocol errors, as the group to which each patient belonged was defined by its location.

Both the Internal Medicine and the General and Digestive Surgery ward are divided into two hospitalization wings. At the time of the study, due to the progressive introduction of the nutrition screening in the HUNSC, one of the two wings of either ward conducted the CIPA screening at hospital admission (intervention wards), while the other did not (control wards, where standard practice was performed).

### Blinding

Due to the nature of the intervention, neither enrolled patients nor the clinical research team could have been blinded to the group they belonged to, although patients were not informed until they signed the consent of their group. The doctors responsible for treatment did not know whether or not patients were enrolled in the study for not to create an intervention bias on the patient. Data analysis is going to be blinded to the intervention assignment.

### Interventions

**Fig. 1:**
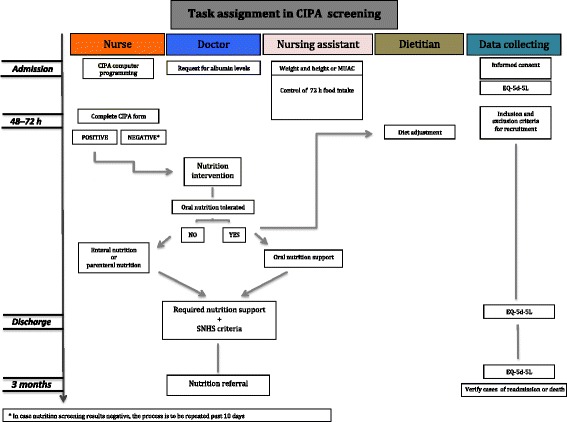
Health care staff’s tasks to accomplish CIPA screening

Patients in the intervention group were subjected to CIPA nutrition screening, which resulted positive if one of the following conditions was met: (1) 72 h food intake control below 50% [12]; (2) serum albumin < 3 g/dL; (3) body mass index (BMI) < 18.5 kg/m^2^ or mid-upper arm circumference (MUAC) ≤ 22.5 cm [13], the latter when the patient could not be weighed and measured. The flow chart in Fig. 1 reflects the health care personnel’s staff’s tasks to accomplish the screening process.

When patients were admitted through the emergency room or on a scheduled basis for any medical reason, the nutrition screening process started on ward admission. Conversely, if patients were admitted for scheduled surgery, nutrition screening began thereafter, when they arrived at the ward.

In case of a positive screening result, the attending doctor would evaluate the adequacy of a nutrition treatment. To this end, if the patient was unable to feed orally, the doctor requested a referral to the Endocrinology and Nutrition Service, whose doctors and nurses were in charge of a nutrition assessment and the administration of the artificial nutrition they considered appropriate. In the case the patient was able to eat by mouth, a dietitian would adjust the diet and monitor the patient’s progress on the third and tenth day, if necessary. In addition, according to the internal protocol, the patient received two specific oral nutrition supplements (ONS) per day, depending on their comorbidities.

As for the control group, routine clinical practice without any nutrition screening was carried out, so that the responsible doctor may have requested the nutrition parameters and considered specific nutrition treatment if appropriate.

**Fig. 2:**
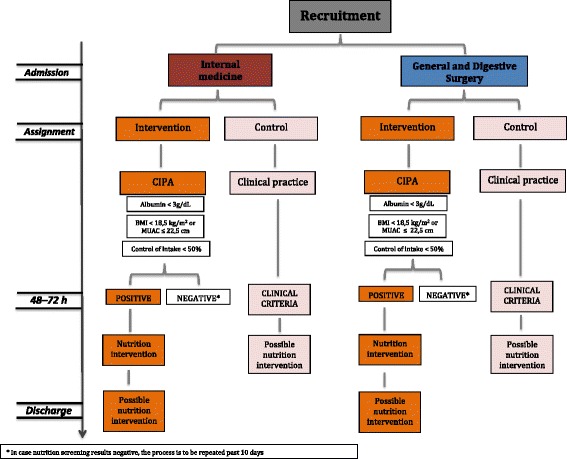
Intervention algorithm

Regardless of the group they belong to, patients with nutrition support during hospitalization may have continued that prescribed support for at least three more months past discharge, provided that the responsible doctor considered the measure appropriate and their pathology was included in the financing list of such treatments of the Spanish National Health System (SNHS). Figure 2 shows the intervention algorithm.

### Ethics

The Scientific and Ethics Committees of the HUNSC approved the study protocol. The study is being performed in accordance with Good Clinical Practice Standards, applicable local regulatory requirements, and the recommendations of the Declaration of Helsinki.

### Endpoints

#### Primary endpoint

The primary endpoint of this study is the difference in mean length of hospital stay between the two compared groups. A difference of 2.6 days is considered clinically significant [13].

#### Secondary endpoints

##### Mortality and morbidity

1. Rate of mortality in in-hospital patients.
2. Rate of mortality in the course of three months post-discharge.
3. Comorbidity will be assessed using the Charlson Index (CI) [14], which comprises 19 categories of the International Classification of Diseases, (10th revision) diagnose codes (ICD-10). It is based on a set of risk factors for the 1-year mortality risk and can be applied to establish the association between the life expectancy of a patient and the resources a health care system must allocate.

##### Complications

1. Readmission rates, i.e., within 3 months post-discharge.
2. Rate of surgical interventions during hospital stay.
3. Incidence of clinical in-hospital complications according to the Classification of Hospital-Acquired Diagnoses (CHADx) [15]. The CHADx tool is a hierarchical classification of encoded diagnoses based on the International Classification of Diseases-10 (ICD-10), which allows quantification of the burden of in-hospital complications.
4. Surgical complications according to the Clavien-Dindo classification, a standardized and internationally validated scoring system. The system allows for the severity of the complication and its interference in the clinical course of the operated patients. The highest grade complication (grades I-V) experienced by each patient was recorded [16].

##### Treatment

1. Rate of prescribed nutrition treatments.
2. Period prior to nutrition treatment.
3. Characteristics of the nutrition treatment (duration, type, indications, prolongation following discharge, changes in the treatment, number of visits of the dietitian, and number of referrals).

##### Quality of life

1. Health-related quality of life (HRQoL) was measured using the EQ-5D-5 L questionnaire. This generic, self-reported description assesses five domains: mobility, self-care, usual activity, pain or discomfort, and anxiety or depression. Each dimension is scored on a scale of 1 to 5, depending on whether the respondent has no problems, slight, moderate, severe, or extreme problems with each dimension. This descriptive system defines 3125 health states. Each health state can then be converted into a single summary index by applying a formula that associates weights to each state, based on evaluations from general population samples, developed by the EuroQoL group. Upon application of these weights, an EQ-5D index score of one represents full health, a score of zero is equivalent to death, and negative scores represent health states worse than death [17].

##### Biochemical determinations

1. Plasma albumin was determined by colorimetry, using a Roche/Hitachi Cobas C 702 Autoanalyzer (Roche Diagnostics). This parameter was requested automatically within 48 h post-admission. Results are expressed in g/dL.

##### Anthropometric determinations

1. BMI: Weight (kg) and height (cm) were obtained with the patient barefoot, dressed in hospital pajamas, using calibrated Seca 220 scales that include a height rod. In the case of patients confined to bed, where weight and height could not be determined, MUAC (cm) was obtained on the patient’s non-dominant arm, at the midpoint between the acromion and olecranon, surrounding the arm with a measuring tape (Seca 201) without pressing the limb. This data was collected by nurses and nursing assistants, when the patient entered the hospital ward, and was then transferred to the electronic medical record (EMR).

##### Health care utilization

Health care utilization includes staff time and material required to implement the malnutrition screening tool CIPA, duration of tube feeding or any other nutritional treatment, visits from other specialists during hospitalization, in-hospital complications, hospital length of stay, and readmissions and other health care contacts within 3 months after discharge.

In addition, the following patient information was collected upon admission: initials, the assigned ward, clinical record, nationality, age, sex, type of admission (emergency or scheduled), date of and diagnosis on admission. Subsequently, the diagnosis-related group (DRG) as well as the severity-based DRG were established.

### Measurement procedures

Data collection is being managed using an electronic case report form (CRF), designed for this study and complemented with EMR information and patient questionnaires conducted by phone 3 months past discharge.

The project data manager compiles all clinical data from the EMR, except for the EQ-5D-5 L questionnaires, which are being collected at hospital admission, discharge, and 3 months post-discharge, and the DRG and severity-based DRG, which are presented by the Management Unit following hospital discharge. The main data and laboratory parameters of included patients are directly recorded in the CRF. The CI and the CHADx and Clavien-Dindo classifications are documented by the medical staff subsequent to the patient’s discharge.

### Statistical methods

#### Sample size calculation

According to the study of Kruizenga et al. [10], 412 patients per arm (a total of 824 patients for this study) are required to detect a difference in hospital stay of 2.6 days, assuming a standard deviation of 8 days in the intervention group and 13.3 days in the control group. Calculations were performed considering a two-tailed power of 90%, an alpha of 0.05, and 10% of potential losses.

#### Statistical analysis

Descriptive data analysis will present qualitative variables as frequencies and percentages, and continuous variables as means, medians, and standard deviations. Normality shall be verified by the Kolmogorov-Smirnov normality test. In bivariate analysis, depending on the nature of the variables, the parametric hypothesis test Student’s *t*-test or the non-parametric Mann-Whitney test will be used. To assess the effects of the intervention, changes in the primary and secondary outcomes will be analyzed by a between-group ANCOVA variance test. The analysis will control for the type, age, and sex of the patient, as well as the responsible medical unit. Patients admitted to Internal Medicine will be categorized into the four groups (1) cardiovascular, (2) neoplastic, (3) infectious, and (4) other patients, whereas patients in the Digestive and General Surgery services will be divided into the categories (1) neoplastic, (2) infectious, (3) obstructive, (4) inflammatory, and (5) others. The economic evaluation and statistical analyses of individual variables will be conducted in accordance with the intention-to-treat principle.

All tests will be two-sided with a type I error of 5%. Statistical analyses will be performed using the Statistical Package for Social Sciences (SPSS v.23, Chicago, IL, USA).

#### Cost-effectiveness analysis

We will analyze the cost and cost-effectiveness of implementing the malnutrition screening tool CIPA versus standard practice in patients admitted to the Internal Medicine and General and Digestive Surgery wards. These analyses will be in line with accepted economic evaluation methods.

The cost will be assessed from a National Health Service perspective and, therefore, only include medical costs incurred by the health care service. Based on data from the hospital as well as the national sources, we will assign a unit cost to each component of health care utilization collected in this trial.

The cost-effectiveness measure will be the incremental cost per quality-adjusted life year (QALY) gained. QALYs will be calculated based on the HRQoL and mortality data collected during the trial. Patient-specific utility profiles will be created assuming a straight-line relation between all patients’ EQ-5D-5 L scores from either follow-up point. The QALYs experienced by each patient from baseline to 3 months will be calculated as the area underneath this profile. We will investigate differences in baseline characteristics and, if necessary, use regression methods to control for them.

Cost-effectiveness will be summarized as the incremental cost-effectiveness ratio (ICER) by dividing the estimated differences in cost by the differences in QALYs. Non-parametric methods to calculate confidence intervals around the ICER, based on bootstrapped estimates of the mean cost and QALY differences, will be used. Further, the bootstrap replications will be used to create a cost-effectiveness acceptability curve, which will reveal the probability that each alternative is cost effective for different values of willingness to pay for an additional QALY. We will also subject the results to extensive, deterministic (one, two-, and multi-way) sensitivity analyses.

#### Duration of fieldwork

The recruitment phase lasted for 15 months. Patients are being followed up for three more months post-discharge. Thereafter, a subsequent phase of data cleansing, identification of unit costs, and data analyses will take place.

#### Monitoring

Trial monitoring is the responsibility of the research team in charge of all quality control activities, assessing adherence to the trial protocol, timely work plan execution, and comprehensiveness of data acquisition and data quality. The databases have been designed to avoid, for each variable, downloading of inappropriate values.

#### Trial status

This study is ongoing, the investigators are still collecting data.

## Discussion

This research project evaluates the effectiveness and cost-effectiveness of implementing a hospital nutrition screening (in this case the CIPA method) in a detailed manner, comprising a large number of variables to analyze the effect of its introduction from different perspectives. On the one hand, clinical variables, such as medical complications during the admission process, mean length of stay, mortality, readmission, or even patient-directed procedures are assessed. On the other hand, data collection includes costs of the visits from dietitians and other skilled health personnel, as well as the costs of nutrition support, not only ONS, but also tube feeding or parenteral feeding, where necessary.

Disease-associated malnutrition is a perfect target for screening due to its high prevalence and the established therapeutic measures that can mitigate its deleterious effects when applied timely, thereby allowing clinical and economic improvement [18–22]. However, there is no ideal method of nutrition screening, as those that best predict the patient’s clinical course are the most complex to perform and require substantial efforts for implementation in hospitals with many patients, as e.g., SGA [7] or NRS2002 [6], while others that are easier and more feasible to carry out, do not predict that course so well. Consequently, the ideal screening system should adapt to the hospital characteristics, predict patient outcome, be workable by most caregivers, and should be inexpensive and not time-consuming [23–25].

The CIPA screening meets all these criteria: (1) it employs tools used in everyday clinical practice that do not require specialized personnel; (2) it is quick to perform; (3) it is objective; (4) it is inexpensive, since the only extra cost is the general determination of serum albumin at hospital admission, which is negligible for a measure with foreseeable, significant savings; (5) it is able to predict the patients’ prognosis, as in a study in 221 non-surgical patients the CIPA screening detected patients with a comparably longer mean length of stay of one week and a higher mortality, similar to the findings of another early study that allowed its development [13, 14].

Ethical constraints on prospective studies of nutrition intervention in malnourished patients resulted in scientific evidence of the benefit of treating these patients primarily from retrospective studies. The outstanding work of Phillipson TJ et al. [20] revealed that treatment with ONS decreased the mean length of stay, readmission rate, and health cost. Likewise, two meta-analyses, recently published by Elia M et al., describe the clinical and economic benefits of ONS treatment in malnourished, in-hospital patients and community settings [21, 22]. Furthermore, a recently published, first prospective, randomized, double-blind study showed that a nutrition supplement decreases the incidence of mortality in in-hospital patients during their stay and up to 3 months post-discharge [23].

Using the SNAQ tool, a study by Kruizenga HM et al. [10] analyzed the cost-effectiveness of the implementation of a nutrition screening at hospital admission. The authors mainly evaluated the decrease in the mean stay as a measure to reduce costs, while taking into account extra expenses due to ONS treatment and dietitian visits, finding the tool to be cost effective primarily in the fragile patient.

Although a poor nutrition status relates to a limited HRQoL both in inpatients as well as non-institutionalized patients, very few studies have assessed HRQoL associated with the nutrition status or treatment in risk patients [26–28], most of them focusing on clinical parameters. The present study includes the analysis of the state of HRQoL by means of the EQ-5D-5 L questionnaire, not only as a generic reference in terms of the association of the nutrition status with the HRQoL, but also because QALYs are the most widely used measure to evaluate the cost-effectiveness of an intervention in the field of health care [29].

The limitations of the present study include the impossibility to perform a double-blind study, as the responsible doctor should know if the patient has screened positive or negative to act on their clinical criterion. In some instances, standard clinical practice may also cause delays in adapting the pattern of nutrition treatment to the screening result, and treatment might be overlooked at hospital discharge even when required by the patient.

It is worth noting that the SNHS only covers the cost of post-discharge nutrition supplements under very specific conditions, e.g., in cancer patients, patients with dysphagia, neurodegenerative diseases, and similar. Consequently, many of the patients who need nutrition support in hospital would not be able to continue after their discharge. For this reason, the number of patients with continued nutrition supplementation after discharge is expected to be rather low in this study.

At the same time, due to training and greater awareness about diagnosis and treatment of malnutrition among the clinical staff of the HUNSC, particularly in the surgical wards, the number of patients excluded for having nutritional support before finalizing the CIPA screening has been higher than expected, which led to an extension of the recruitment period.

Therefore, with the scientific rationale in favor of a nutrition management of malnourished patients and with tools for early detection of this condition at hand, it is foreseeable that the implementation of a cheap and simple hospital nutrition screening results in a cost-effective intervention. However, properly designed and methodologically unbiased studies are required to evaluate this relationship. Given the scarcity of literature in regard to this type of evaluations, we hope to fill the evidence gap between hospital nutrition screening and health care cost savings with this study, and, if proven cost effective, promote the universal implementation of the CIPA screening system in hospitals through appropriate political strategies.

## Abbreviations

BMI: Body mass index
CHADx: Classification of hospital-acquired diagnoses
CI: Charlson index
CIPA: Control of food intake, protein and anthropometry
CRF: Case report form
DRG: Diagnosis-related group
EMR: Electronic medical record
HM: Hospital malnutrition
HRQoL: Health-related quality of life
HUNSC: Hospital Universitario Nuestra Señora de Candelaria
ICD-10: International classification of diseases (10th revision)
ICER: Incremental cost-effectiveness ratio
MUAC: Mid-upper arm circumference
NRS2002: Nutritional risk screening 2002
ONS: Oral Nutrition supplements
QALYs: Quality-adjusted life years
SGA: Subjective global assessment
SNAQ: Short nutritional assessment questionnaire
SNHS: Spanish national health system
